# Superconductor qubits hamiltonian approximations effect on quantum state evolution and control

**DOI:** 10.1038/s41598-021-92290-0

**Published:** 2021-06-17

**Authors:** Javad Sharifi

**Affiliations:** Electrical and Computer Engineering Department, Qom University of Technology, Qom, 37181-46645 Iran

**Keywords:** Quantum physics, Qubits

## Abstract

Microwave IQ-mixer controllers are designed for the three approximated Hamiltonians of charge, phase and flux qubits and the controllers are exerted both on approximate and precise quantum system models. The controlled qubits are for the implementation of the two quantum-gates with these three fundamental types of qubits, Quantum NOT-gate and Hadamard-gate. In the charge-qubit, for implementation of both gates, in the approximated and precise model, we observed different controlled trajectories. But fortunately, applying the controller designed for the approximated system over the precise system leads to the passing of the quantum state from the desired state sooner that the expected time. Phase-qubit and flux qubit have similar behaviour under the control system action. In both of them, the implementation of NOT-gate operation led to same trajectories which arrive at final goal state at different times. But in both of those two qubits for implementation of Hadamard-gate, desired trajectory and precise trajectory have some angle of deviation, then by exerting the approximated design controller to precise system, it caused the quantum state to approach the goal state for Hadamard gate implementation, and since the quantum state does not completely reach the goal state, we can not obtain very high gate fidelity.

## Introduction

Quantum engineering harnesses the quantum state, estimates and eliminates environmental noise on open quantum systems, corrects quantum errors, for the sake of quantum computing. Excellent quantum engineering framework researches are carried for quantum computing applications with trapped ions^1,2^, spin qubit control^3–5^, quantum optical control of semiconductor quantum dots^6–8^, superconducting qubits^9–12^, etc. Among different approaches to quantum computers, since superconductor circuits can be fabricated and controled based on the current technologies, this has impelled the attention of researches^13–17^ and IC makers, IT companies such as IBM, Intel, Google, Microsoft, etc to make quantum processor with exceptional computational performance with respect to conventional processors. Achieving this goal, the quantum sate of each qubit as fundamental building block of quantum computation must be controlled precisely toward the desired state and remain stable at that state.

For simulations, the QuTiP^18^, NumPy^19^, Matplolib^20^ Python packages are applied and all of data are from a table at^21^. At first, we introduce the Hamiltonian of basic superconductor qubits and their approximations, then by simulations show that the two systems, rotate along different trajectories on Bloch sphere and with different quantum observable expectations, then we endevour to design microwave controller for approximated system and show that this control leads to wrong results with respect to desired trajectories. Finally, we design Lyapunov control for general charge-phase-flux nonlinear circuit which converges to the desired state. By the Schrödinger equation $i\hslash \frac{d}{dt}|\psi \rangle =H|\psi \rangle$ and its unitary evolution

1 $$\begin{aligned} U_{t}=\mathrm {{exp}}\big (\frac{{-i}}{\hslash }\int _0^t H(t)dt\big ) \end{aligned}$$

then the solution is $|\psi _{t}\rangle =e^{\frac{-i}{\hslash }Ht}|\psi _{0}\rangle =U_{t}|\psi _{0}\rangle$ , let *X* be a hermitian observable, the evolution of observable at time *t* is $X_{t}=U^{\dag }_{t}XU_{t}$ and the mean value of a observable based on density matrix $\rho _{t}=|\psi _{t}\rangle \langle \psi _{t}|$ is $\langle X \rangle =\mathrm {Tr}(\rho _{t} X)$. The quantum state on Bloch sphere is $|\psi \rangle =\cos (\frac{1}{2}\theta )|0 \rangle +e^{i\phi }\mathrm {sin}(\frac{ 1}{2}\theta )|1 \rangle$ where $|0 \rangle$ and $|1 \rangle$ are qubit eigenbasis or in density matrix form as:

$$\begin{aligned} { \rho _{t}=\frac{1}{2}\begin{pmatrix} 1+z&{}x-iy\\ x+iy&{}1-z \end{pmatrix}} \end{aligned}$$

where $(x,y,z)=\vec {r}=(\mathrm {sin}(\theta )\cos (\phi ),\mathrm {sin}(\theta )\mathrm {sin}(\phi ),\cos (\theta ))$ is vector on spherical coordinate, in this form the quantum system is governed by master equation ${\dot{\rho }}_{t}=\frac{i}{\hslash }[\rho _{t},H]$. The unitary rotation operator for transition of quantum states is

2 $$ U_{\mathrm {rot}}={\mathbf {R}}_{{\hat{n}}}(\alpha )=e^{-i\frac{\alpha }{2}{\hat{n}}.\vec {\sigma }}= {\sin}(\frac{\alpha }{2})I-i\cos (\frac{\alpha }{2}){\hat{n}}.\vec{\sigma } $$

with $\vec {\sigma }=\sigma _{x}{\hat{x}}+\sigma _{y}{\hat{y}}+\sigma _{z}{\hat{z}}$ and $\sigma _{x},\sigma _{y},\sigma _{z}$ are Pauli matrices and ${\hat{n}}=n_{x}{\hat{x}}+n_{y}{\hat{y}}+n_{z}{\hat{z}}$ is the rotation axis and $\alpha =\omega _{q} t$ is rotation angle, $\omega _{q}$ is the angular speed of quantum state vector on Bloch sphere.

## Superconductor qubits evolution

**Figure 1:**
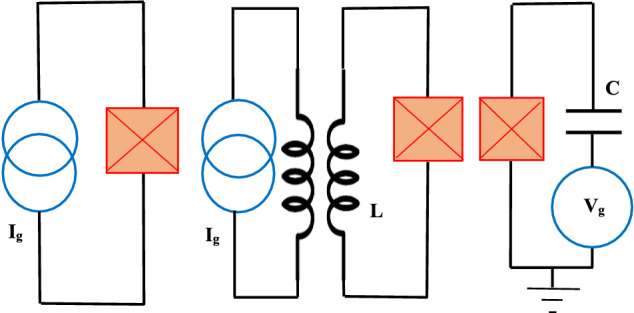
Basic superconductor qubits: phase qubit (left), flux qubit (middle) and charge qubit (right).

The fundamental superconductor circuits are depicted in Fig. 1.

**Figure 2:**
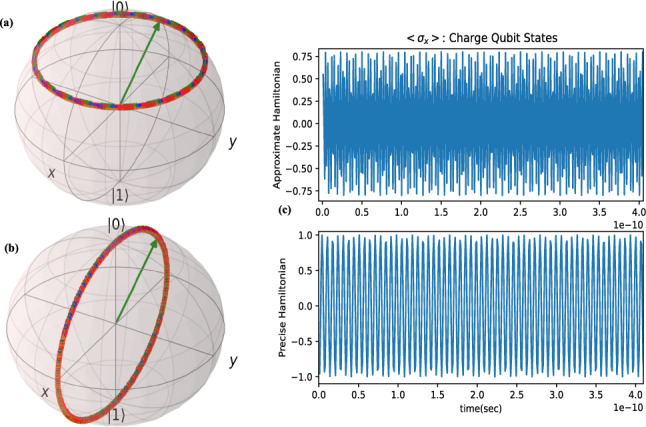
Charge qubit state transition without external drive voltage $V_{g}=1$mV, $C_{g}=0.68$fF and $E_{J}=0.018E_{c}$, $E_{c}=7.55\times 10^{-23}$J: quantum state on Bloch sphere with approximate Hamiltonian (**a**) and precise Hamiltonian (**b**) and the mean value of sates(c). For simulations, QuTiP^18^ and Matplotlib^20^ Python packages are used.

**Charge qubit** or C-JJ circuit precise Hamiltonian is^22^: ${\hat{H}}_{pre}=E_{c}(\hat{{N}}-n_{g})^{2}-E_{J}\cos ({\hat{\phi }})$, in which phase operator ${\hat{\phi }}$ and charge operator $\hat{{N}}=-i\hslash \frac{\partial }{\partial {{\hat{\phi }}}}$ satisfy the commutation relation $[{\hat{\phi }},\hat{{N}}]=i\hslash$. $E_{c}=(2e)^2/2(C+C_{g})$ are the charging energy of one 2e Cooper pair in which e is electron charge,$E_{J}=\frac{\hslash }{2e}I_{c}$ is Josephson energy, and $C_{g}$ and $I_{c}$ are Josephson Junction (JJ) capacitor and Josephson critical current, and $n_{g}=\frac{1}{2}C_{g}V_{g}/e$ is the voltage-induced charge on the capacitor *C*, and it is the control parameter. The approximate Hamiltonian with forth order approximation of cosine term and after second quantization is ${\hat{H}}_{app}=\hbar \omega _{q} a^{\dagger }a+\frac{\beta }{2}a^{\dagger }a^{\dagger }aa$ and resembles a Duffing oscillator. The parameters $\omega _{q}$ and $\beta$ are angular frequency in rad/sec and energy in Joule, respectively. The number operator and phase operator for qubits are set as $\hat{{N}}=in_{zpf}(a-a^{\dagger })$ and ${\hat{\phi }}=\phi _{zpf}(a+a^{\dagger })$ where $n_{zpf}=(E_{L_{J0}}/32E_{c})^\frac{1}{4}$ and $\phi _{zpf}=(2E_{c}/E_{L_{J0}})^\frac{1}{4}$ are number and phase zero-point-fluctuation and $E_{L_{J0}}=\hslash ^2/(4e^2L_{J0})$. For qubits, the creation and annihilation operators are respectively the raising and lowering ladder operators, i.e. $a^{\dagger }=\sigma _{+} , a=\sigma _{-}$. By assumption $E_{c}>>E_{J}$ the Hamiltonian simplifies to^23^: ${\hat{H}}_{app}=E_{c}(\frac{1}{2}-n_{g})\sigma _{z}+\frac{1}{2}E_{J}\sigma _{x}$ and then the quantum state is evolveed by equation $|\psi \rangle =e^{-\frac{it}{\hslash }(E_{c}(\frac{1}{2}-n_{g})\sigma _{z}+\frac{1}{2}E_{J}\sigma _{x})}|\psi _{0}\rangle$ which by comparison to unitary rotation operator, this corresponds to the rotation around an axis on x-z plane on Bloch sphere as the simulation results of Fig. 2a confirm it. To obtain the evolution difference for precise Hamiltonian and approximated Hamiltonin let us compute the precise value of $\cos ({\hat{\phi }})$ for ${\hat{\phi }}=\phi _{zpf}\sigma _{x}$, in this situation with Cayley-Hamilton theorem, we will obtain $\cos ({\hat{\phi }})=\cos (\phi _{0})I$. Furthermore, for ${\hat{N}}=n_{zpf}\sigma _{y}$ the term $({\hat{N}}-n_{g})^{2}$ of precise Hamiltonian is $(n_{g}^{2}+n_{zpf}^{2})I-2n_{zpg}n_{g}\sigma _{y}$, then the charge-qubit precise Hamiltonain is:

3 $$\begin{aligned} { {\hat{H}}_{pre}=(E_{c}(n_{g}^{2}+n_{zpf}^{2})-E_{J}\cos (\phi _{zpf}))I-2E_{c}n_{zpg}n_{g}\sigma _{y} } \end{aligned}$$

The simulation of state evolution with exact Hamiltonian is shown in Fig. 2b. We mention that for zero deriving voltage of charge qubit (JJ-C), the state vector will remain constant to initial state on the surface of Bloch sphere. In Fig. 2c, the expectation value of a Pauli matrix , i.e. $\langle \sigma _{x} \rangle$ is plotted.

**Phase qubit** is depicted in right section of Fig. 1 and obeys the relation^22^: ${\hat{H}}_{pre}=E_{c}\hat{{N}}^{2}-E_{J}\cos ({\hat{\phi }})-\frac{\hslash }{2e}I_{g}{\hat{\phi }}$ where $I_{g}$ is the control current. After some simple calculations, we will obtain the precise Hamiltonian of phase-qubit as

4 $$\begin{aligned} { {\hat{H}}_{pre}=(E_{c}n_{zpf}^{2}-E_{J}\cos (\phi _{zpf}))I-\frac{\hslash }{2e}I_{g}\phi _{zpf}\sigma _{x} } \end{aligned}$$

**Figure 3:**
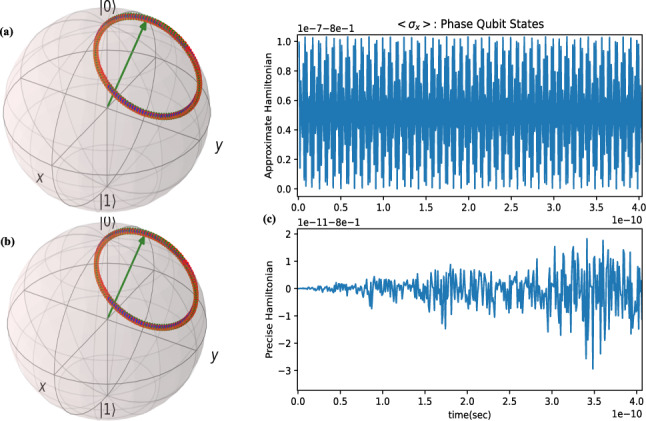
Phase qubit state evolutiopn($E_{J}=3.266\times 10^{-23}$, $E_{c}=10^{-4}E_{J}$, $\phi _{zpf}=0.0398$, $I_{g}=1$ mA): state evolution on Bloch Sphere with approximate Hamiltonian (**a**) and precise Hamiltonian (**b**) and the mean value of sates(c). For simulations, QuTiP^18^ and Matplotlib^20^ Python packages are used.

The approximate Hamiltonian is ${\hat{H}}_{app}=-\frac{1}{2}E_{c}\sigma _{z}+(\frac{1}{2}E_{J}-\frac{\hslash }{2e}\phi _{zpf}I_{g})\sigma _{x}$. Simulations of phase qubit evolution both for approximate and precise Hamiltonian is depicted in Fig. 3a,b respectively, which shows the same result trajectory; but, the expectation value of quantum observable $\langle \sigma _{x}\rangle$ is completely different for the two Hamiltonians (Fig. 3c).

**Flux qubit** (L-JJ) is depicted in middle of Fig. 1 and obeys the relation^22^: ${\hat{H}}_{pre}=E_{c}\hat{{N}}^{2}-E_{J}\cos ({\hat{\phi }})+\frac{1}{2}E_{L} ({\hat{\phi }}-\phi _{e})^2$ that $E_{L}=\hslash ^2/(4e^2L)$ and $\phi _{e}$ is the control parameter and we compute it as:

5 $$\begin{aligned} { {\hat{H}}_{pre}=(E_{c}n_{zpf}^{2}-E_{J}\cos (\phi _{zpf}))I-E_{L}\phi _{zpf}\phi _{e}\sigma _{x} } \end{aligned}$$

**Figure 4:**
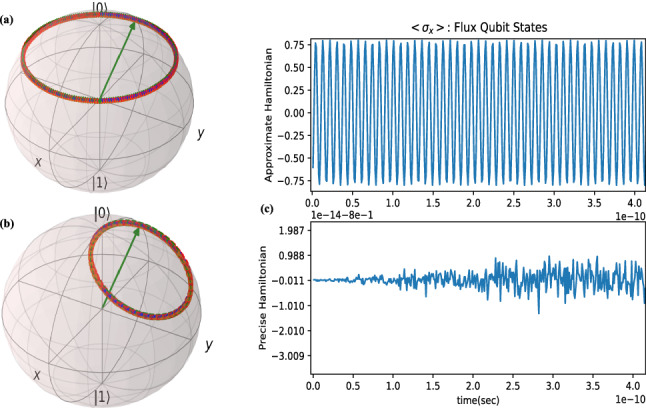
Flux qubit state transition: State transition on Bloch sphere with approximate Hamiltonian (Top) and precise Hamiltonian (Bottom) and the mean value of sates(c): $E_{J}=6.017\times 10^{-23}$J , $E_{c}=1.711\times 10^{-23}$J. For simulations, QuTiP^18^ and Matplotlib^20^ Python packages are used.

Approximate Hamiltonian is ${\hat{H}}_{app}=-\frac{1}{2}E_{c}\sigma _{z}+(\frac{1}{2}E_{J}-E_{L}\phi _{zpf}\phi _{e})\sigma _{x}$. For approximate Hamiltonian, the trajectory of quantum state on Bloch sphere rotate around z-axis (Fig. 4a) but trajectory rotation change for precise model of Hamiltonian (Fig. 4b) and also the expectation $\langle \sigma _{x} \rangle$ of both Hamiltonian is different (Fig. 4c). We found from these simulations that approximating the superconducting qubits leads to wrong state evolution and considerable error in expectation of quantum observable.

## Effect on superconductor qubits control

By the equation of Hamiltonian in previous section, it is figured out that by constant control signal, it is not possible to control quantum state vector over whole space through rotation of quantum state on three directions; for example, by approximate Hamiltonian of charge qubit we can control the rotation of state around z-axis. one approach to overcome this shortage is by microwave driving oscillator (Rabi driving)^24–27^ which is practically produced by arbitrary wave generator (AWG).

**Charge qubit control** Here, we drive the charge qubit by the signal which contains both Rabi drive and non-oscillating dc signal to fully control the quantum state on three rotation directions; assume the signal as $V_{g}(t)=V_{ac}+V_{dc}=V_{0}s(t)\mathrm {sin}(\omega _{c}t+\lambda )+V_{g0}$ which $\omega _{c}=2\pi f_{c}$ is control signal frequency in rad/sec ($f_{c}$ in Hz), the initial non-rotating frame control Hamiltonian is ${\hat{H}}_{c}=-E_{c}n_{g}\sigma _{z}$ then the Hamiltonian of rotating part is given by ${\hat{H}}_{rf}=U^{\dag }_{0}kV_{0}s(t)\mathrm {sin}(\omega _{c}t+\lambda )\sigma _{z}U_{0}$ where $U_{0}=e^{-\frac{it}{\hslash }(\frac{1}{2}E_{c}\sigma _{z}+\frac{1}{2}E_{J}\sigma _{x})}=e^{-\frac{i}{2}\omega _{z}t\sigma _{z}-\frac{i}{2}\omega _{x}t\sigma _{x}}$ and $k=(-\frac{1}{2e}C_{g}E_{c})$, multiplication in ${\hat{H}}_{rf}$ has sine and cosine terms with frequencies $\omega _{c}-(\omega _{z}-\omega _{x})$ , $\omega _{c}+\omega _{z}+\omega _{x}$ , $\omega _{c}+\omega _{z}-\omega _{x}$ , $\omega _{c}-\omega _{z}-\omega _{x}$ and since for charge qubit $\omega _{z}>>\omega _{x}$ by rotating wave approximation, only first term is held and the other three fast rotating terms and constant offset can be ignored, then the rf-controlled Hamiltonian is:

6 $$\begin{aligned} \begin{aligned} {\hat{H}}_{rf}=&\frac{_1}{^{8}}kV_{0}s(t)(\mathrm {sin}(\delta \omega t+\lambda )\sigma _{x}-2\cos (\delta \omega t+\lambda )\sigma _{y}\\&+\mathrm {sin}(\delta \omega t+\lambda )\sigma _{z}) \end{aligned} \end{aligned}$$

which in this equation $\delta \omega =\omega _{c}-(\omega _{z}-\omega _{x})$, if we set $\delta \omega =0$, only the phase difference between radio frequency with frequency $\omega _{x}$ plays control role and the rf-controlled Hamiltonian simplifies to ${\hat{H}}_{rf}=\frac{1}{8}kV_{0}s(t)(Q\sigma _{x}-2I\sigma _{y}+Q\sigma _{z})$ which $I=\mathrm {{cos}}(\lambda ),Q=\mathrm {{sin}}(\lambda )$, this method is the method of IQ-mixer. To increase the control signal degree of freedom, a constant voltage pulse can be added to the microwave rotating voltage which leads to the total control propagator operator as:

7 $$\begin{aligned} \begin{aligned} U_{c}(t)=e^{\frac{-ik}{\hslash }(\frac{V_{0}}{8}\gamma (t)Q\sigma _{x}-\frac{V_{0}}{4}\gamma (t)I\sigma _{y}+(\frac{V_{0}}{8}\gamma (t)Q+V_{g0}t)\sigma _{z})} \end{aligned} \end{aligned}$$

$\gamma (t)=\int _0^t s(t)dt$, for simplicity let set $s(t)=1$ and then $\gamma (t)=t$, hence by comparison of rotation propagator of Eq. (2), it obtains: $\omega _{q}n_{x}=\frac{1}{4\hslash }kV_{0}Q , \omega _{q}n_{y}=-\frac{1}{2\hslash }kV_{0}I , \omega _{q}n_{z}=\frac{1}{4\hslash }kV_{0}Q+\frac{2}{\hslash }kV_{g0}$ then the whole signal parameters are: $\lambda =\mathrm {{tan}}^{-1}(-\frac{2n_{x}}{n_{y}}) , V_{0}=\frac{4\hslash \omega _{q} n_{x}}{kQ} , V_{g0}=\frac{\hslash \omega _{q}}{2k}(n_{z}-n_{x})$. Now, consider the control signal which aims at transiting initial quantum state $|\psi _{0} \rangle$ to final quantum state $|\psi _{f} \rangle$ at finite time $t_{f}$, one approach is to rotate the initial vector to final vector around the around the bisector of two unit vectors $\vec {r_{0}},\vec {r_{f}}$ by angle $\pm \pi$, then:

8 $$\begin{aligned} \begin{pmatrix} {\hat{n}}\\ \alpha \end{pmatrix} = \begin{pmatrix} \frac{\vec {r_{0}}+\vec {r_{f}}}{|\vec {r_{0}}+\vec {r_{f}}|}\\ \pm \pi \end{pmatrix} \end{aligned}$$

**Figure 5:**
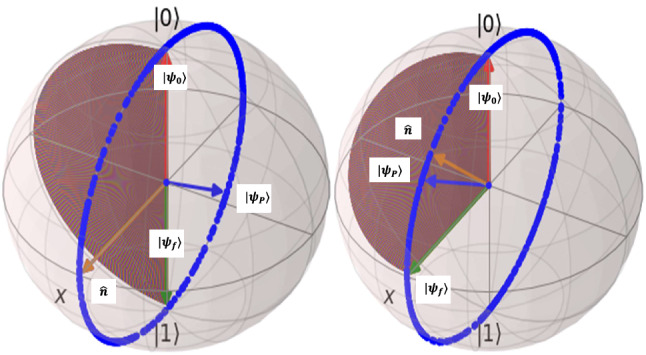
Quantum state control of superconductor charge qubit for implementation of quantum NOT-gate(left) and Hadamard gate(right): $|\psi _{0}\rangle$ is initial state $|\psi _{f}\rangle$ is final desired state. Desired trajectory is depicted with brown vectors. By exerting the designed controller for approximate system to precise system, quantum state vector rotates through blue trajectory and reaches to state $|\psi _{P}\rangle$ at $t_{f}=1$nsec; however, the quantum state seems to reach the desired state (Brown vector, $|1\rangle$ for NOT-gate and $|+\rangle$ for Hadamard-gate) at a earlier time $t_{*}<t_{f}$. For simulations, QuTiP^18^ Python package is used.

As a quantum computing instance, for implementation of quantum NOT-gate, we must design quantum control signal in such a way that trasfer happens from the state $|0\rangle$ to state $|1\rangle$ and conversely. Then for example consider the following initial and final states for NOT implementation: $|\psi _{0}\rangle =|0\rangle , |\psi _{f}\rangle =|1\rangle$, then $\vec {r_{0}}=(0,0,1) , \vec {r_{f}}=(0,0,-1)$ then ${\hat{n}}=(1,0,0)$ and by $\alpha =\pi , t_{f}=1$nsec, we obtain : $\lambda =-\frac{\pi }{2}$ rad , $V_{0}=8.27\mu$V , $V_{g0}=0$V , $\omega _{c}=15935468926$ rad/sec (or $f_{c}=2.53621$GHz). In Fig. 5 the initial state, final state and the two trajectories of states on Bloch sphere are depicted both for NOT-gate (left) and Hadamard-gate(right). By designing controller for approximated Hamiltonian charge qubit, the quantum state on approximated system along brown-vectors trajectory reachs to final desired state at time $t_{f}=1$nsec. But, the result of control signal on this actual precise system along blue trajectory reaches to state $|\psi _{P}\rangle$ which has significant error from final state; however, fortunately, in an earlier time $t_{*}<t_{f}$ along blue-trajectory, the system passes from final state, but for final time $t_{f}$ it reaches to a wrong state $|\psi _{P}\rangle$. It is the fortune of previous researches that had using approximate model, becuase despite of the different trajectories in some time in charge qubit, they arrive at the right final state. Hence, by some manipulations they had successful achievement to implement the quantum NOT-gate based on charge-qubit circuit. However, during the designed final time $t_{f}$ the actual system state of charge qubit pass from desired state $|1\rangle$ and stop at wrong stste. The Hadamard-gate rotate the quantum states $|0\rangle , |1\rangle$ to $|+\rangle =\frac{1}{2}(|0\rangle +|1\rangle ) , |-\rangle =\frac{1}{2}(|0\rangle -|1\rangle )$ states respectively. For simulations we will consider the case $|\psi _{0}\rangle =|0\rangle , |\psi _{f}\rangle =|+\rangle$, then it leads to $\vec {r_{0}}=(0,0,1) , \vec {r_{f}}=(1,0,0)$ then ${\hat{n}}=\frac{1}{\sqrt{2}}(1,0,1)$ and $V_{0}=5.848\mu$V and all the other parameters are the same as NOT-gate implementation. In right of Fig. 5 the simulation results leads to same explanation as NOT-gate of charge qubit; hence, based on approximate Hamiltonian of charge qubit, we successfullty implemented the NOT and Hadamard gate, the desgined time $t_{f}$ is not true for precise model and earlier than this time in practice; therefore, the control signals must be off. However, we can reach final state for quantum gate implementation in different control signal final time but the quantum states trajectories for these types of qubits are completely different.

At time $t_{f}$ the control signal sets to zero, and hence the quantum state remains at the final state, but if someone uses the approximate model, the control signal must set off at time $t_{*}<t_{f}$. To obtain this time, an optimization problem must be solved for each quantum-gate implementation. Here we formulate this optimization problem; however, we do not solve it, since this is not the aim of this reserach. Based on Eq. (3), control Hamiltonian for precise system is ${\hat{H}}_{c_{pre}}=E_{c}n_{g}^{2}I-2E_{c}n_{zpg}n_{g}\sigma _{y}$ and the free unitary propagator is $U_{0pre}=exp(-\frac{i}{\hbar }((E_{c}n_{zpf}^{2})-E_{J}\cos (\phi _{zpf}))I)$ then the RF-Hamiltonian can be computed as ${\hat{H}}_{rf_{pre}}=U^{\dag }_{0pre}{\hat{H}}_{c_{pre}}U_{0pre}$ and since the propagator $U_{0pre}$ is diagonal and unitary, then ${\hat{H}}_{rf_{pre}}={\hat{H}}_{c_{pre}}$ and this leads to control propagator $U_{c_{pre}}=e^{-\frac{i}{\hbar }{\hat{H}}_{rf_{pre}}t}=e^{-\frac{i}{\hbar }{\hat{H}}_{c_{pre}}}=e^{-\frac{i}{\hbar }(E_{c}n_{g}^{2}I-2E_{c}n_{zpg}n_{g}\sigma _{y})t}$, then by some calculations and also using the Eq. (2) we will obtain:

9 $$\begin{aligned} {U_{c_{pre}}=\big (\cos (\frac{2}{\hbar }E_{c}n_{zpf}n_{g}t)I+i \mathrm {sin}(\frac{2}{\hbar }E_{c}n_{zpf}n_{g}t)\sigma _{y}\big )e^{-\frac{i}{\hbar }E_{c}n^2_{g}t} } \end{aligned}$$

Then to solve the problem to find the time that quantum state reaches the desired state, based on this final control propagator for precise charge-qubit it must be used for evolution of controlled quantum state from initial state $|\psi _{0}\rangle$ to final state $|\psi _{f}\rangle$ at time $t_{*}$. For example for a NOT-gate let set $|\psi _{0}\rangle =|0\rangle , |\psi _{f}\rangle =|1\rangle$ then we must solve the quantum state-vector equation $U_{c_{pre}}|0\rangle = |1\rangle$ for NOT-gate and $U_{c_{pre}}|0\rangle = |+\rangle$ for Hadamard-gate.

**Phase qubit control** with the same strategy for phase qubit we can design controller for approximated system, for this consider ${\hat{H}}_{c}=-\frac{\hslash }{2e}\phi _{zpf}I_{g}\sigma _{x}$ that as $I_{g}(t)=I_{ac}+I_{dc}=I_{0}s(t)\mathrm {sin}(\omega _{c}t+\lambda )+I_{g0}$, the rf-controlled Hamiltonian as ${\hat{H}}_{rf}=U^{\dag }_{0}kI_{0}s(t)\mathrm {sin}(\omega _{c}t+\lambda )\sigma _{x}U_{0}$ that here $k=-\frac{\hslash }{2e}\phi _{zpf}$ and $U_{0}=e^{-\frac{it}{\hslash }(-\frac{1}{2}E_{c}\sigma _{z}+\frac{1}{2}E_{J}\sigma _{x})}=e^{\frac{i}{2}\omega _{z}t\sigma _{z}-\frac{i}{2}\omega _{x}t\sigma _{x}}$, with similar approach to charge qubit, multiplication ${\hat{H}}_{rf}$ has sine and cosine terms with frequencies $\omega _{c}-(\omega _{x}-\omega _{z})$ , $\omega _{c}+\omega _{x}+\omega _{z}$ , $\omega _{c}+\omega _{x}-\omega _{z}$ , $\omega _{c}-\omega _{x}-\omega _{z}$ and since for phase qubit $\omega _{x}>>\omega _{z}$ by rotating wave approximation, only first term is hold and other three fast rotating terms and constant offset can be ignored and setting $\delta \omega =\omega _{c}-(\omega _{x}-\omega _{z})=0$, then the rf-controlled Hamiltonian is:

10 $$\begin{aligned} \begin{aligned} {\hat{H}}_{rf}=\frac{_1}{^{16}}kI_{0}s(t)(Q\sigma _{x}-4I\sigma _{y}-4Q\sigma _{z}) \end{aligned} \end{aligned}$$

**Figure 6:**
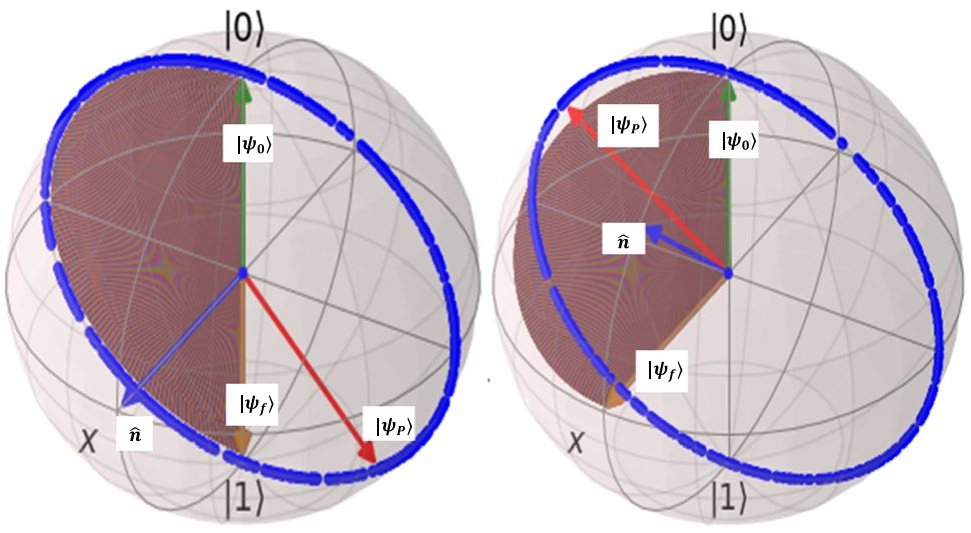
Quantum state control of superconductor phase qubit for implementation of quantum NOT-gate(left) and Hadamard gate(right): $|\psi _{0}\rangle$ is initial state, $|\psi _{f}\rangle$ is final desired state. Desired trajectory is depicted in brown. Exerting the designed controller to main system move through blue trajectory and reaches to state $|\psi _{P}\rangle$. For Not-gate, through blue trajectory the quantum state pass from desired state $|0\rangle$ at some time $t_{*}<t_{f}$ and for Hadamrd gate at some other time the quantum state through blue trajectory passes from a vicinity of desired state $|+\rangle$. For simulations, QuTiP^18^ Python package is used.

By adding constant dc current pulse to change the total control propagator operator to:

11 $$\begin{aligned} \begin{aligned} U_{c}(t)=e^{\frac{-ik}{\hslash }(\frac{I_{0}}{16}\gamma (t)Q\sigma _{x}-\frac{I_{0}}{4}\gamma (t)I\sigma _{y}+(-\frac{I_{0}}{4}\gamma (t)I+I_{g0}t)\sigma _{z})} \end{aligned} \end{aligned}$$

comparing to rotation propagator parameters of Eq. (2), it obtains: $\omega _{q}n_{x}=\frac{1}{8\hslash }kI_{0}Q , \omega _{q}n_{y}=-\frac{1}{2\hslash }kI_{0}I , \omega _{q}n_{z}=-\frac{1}{2\hslash }kI_{0}Q+\frac{2}{\hslash }kI_{g0}$ then the whole signal parameters are: $\lambda =\mathrm {{tan}}^{-1}(-\frac{4n_{x}}{n_{y}}) , I_{0}=\frac{8\hslash \omega _{q} n_{x}}{kQ} , I_{g0}=\frac{\hslash \omega _{q}}{2k}(n_{z}+4n_{x})$. For this qubit type $\lambda =-\frac{\pi }{2}$ rad, $\omega _{c}=31104806366$ rad/sec(or $f_{c}=4.95$GHz) in general and for NOT-gate we have the current parameters $I_{0}=67.7$nA , $I_{g0}=-16.93$nA , and for Hadamard-gate we have current parameters as $I_{0}=47.88$nA , $I_{g0}=-14.96$nA. Also we set the control final time equal to one nano-second. Figure 6 shows the result of simulations for this selection of initial and final states. The Left of this figure is for NOT gate and right is for Hadamard gate. For the NOT-gate implementation, both desired trajectory(brown) is alonside with precise Hamiltonian trajectory(blue), but in exterting of approximate based controller to precise system cause that quantum state rotate several around an ellipse and then rest at state $|\psi _{P}\rangle$ which passes from the desired state at final stop time $t_{f}$. To obtain the true time for precise system which is under approximate control, similar to charge-qubit the control propagator must be computed and the optimization problem must be solved. The right Bloch sphere of this figure is for Hadamard-gate which we found that the desired trajectory and precise trajectory had some angle of deviation. in this gate, the precise trajectory based on approximate control never touches the desired state $|+\rangle$ but in some time $t_{*}<t_{f}$ comes to the vicinity of desired state, in this case also we must optimize a cost fuction based on controlled propagator and find the time and as a consequense the relevant state state $|\psi _{t_{*}}\rangle$, then we must minimize the lenght of quantum state-vector $\vec {V}_{error}=U_{c_{pre}}|\psi _{0}\rangle - |\psi _{f}\rangle$, and define the cost function $J=||\vec {V}_{error}||^2$. This cost function must be optimized over time. For each single qubit gate the propagator is the same but the initial and final states differ. Since this optimized state for Hadamard gate is not the same as final desired state; then, the approximate control for phase-qubit for implementation of Hadamard gate can not lead to high gate fidelities. The controlled propagator here by using design stategy of charge qubit and by using Eqs. 4 and  2 it will be obtained as:

12 $$\begin{aligned} {U_{c_{pre}}=\cos (\frac{1}{2e}\phi _{zpf}I_{g}t)I+i \mathrm {sin}(\frac{1}{2e}\phi _{zpf}I_{e}t)\sigma _{x} } \end{aligned}$$

**Flux qubit control** The flux qubit approximate Hamiltonian is completely similar to phase qubit approximate Hamiltonian with the difference is that control derive is magnetic flux; hence, by considering ${\hat{H}}_{c}=-E_{L}\phi _{zpf}\phi _{e}\sigma _{x}$ that as $\phi _{e}(t)=\phi _{ac}+\phi _{dc}=\phi _{0}s(t)\mathrm {sin}(\omega _{c}t+\lambda )+\phi _{dc}$, the rf-controlled Hamiltonian as ${\hat{H}}_{rf}=U^{\dag }_{0}k\phi _{0}s(t)\mathrm {sin}(\omega _{c}t+\lambda )\sigma _{x}U_{0}$ in which $U_{0}=e^{-\frac{it}{\hslash }(\frac{1}{2}E_{c}\sigma _{z}+\frac{1}{2}E_{J}\sigma _{x})}=e^{-\frac{i}{2}\omega _{z}t\sigma _{z}-\frac{i}{2}\omega _{x}t\sigma _{x}}$ and $k=-E_{L}\phi _{zpf}$, by simplification of sine, cosine products, we will have the frequencies $\omega _{c}-(\omega _{x}-\omega _{z})$ , $\omega _{c}+\omega _{x}+\omega _{z}$ , $\omega _{c}+\omega _{x}-\omega _{z}$ , $\omega _{c}-\omega _{x}-\omega _{z}$ and since for flux qubit $\omega _{x}>\omega _{z}$ by rotating wave approximation, only first term is held and the other three fast rotating terms and constant offset can be ignored and setting $\delta \omega =\omega _{c}-(\omega _{x}-\omega _{z})=0$, then the rf-controlled Hamiltonian is:

13 $$\begin{aligned} \begin{aligned} {\hat{H}}_{rf}=\frac{_1}{^{16}}k\phi _{0}s(t)(Q\sigma _{x}-4I\sigma _{y}-4Q\sigma _{z}) \end{aligned} \end{aligned}$$

By adding constant dc flux pulse to change the total control propagator operator to:

14 $$\begin{aligned} \begin{aligned} U_{c}(t)=e^{\frac{-ik}{\hslash }(\frac{\phi _{0}}{16}\gamma (t)Q\sigma _{x}-\frac{\phi _{0}}{4}\gamma (t)I\sigma _{y}+(-\frac{\phi _{0}}{4}\gamma (t)I+\phi _{dc}t)\sigma _{z})} \end{aligned} \end{aligned}$$

**Figure 7:**
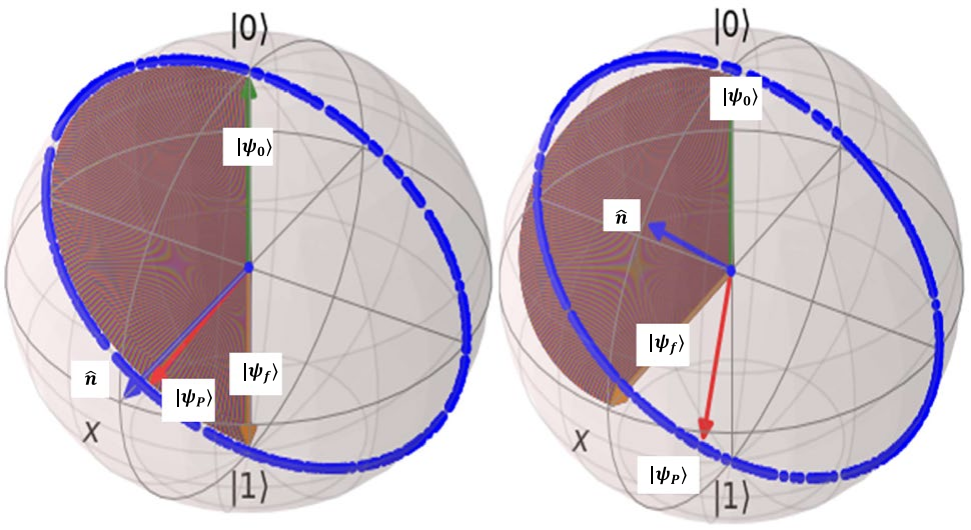
Quantum state control of superconductor flux qubit for implementation of quantum NOT-gate(left) and Hadamard gate(right): $|\psi _{0}\rangle$ is initial state $|\psi _{f}\rangle$ is final desired state. Desired trajectory is depicted in brown. Exerting the designed controller to main system moves through blue trajectory and reachs state $|\psi _{P}\rangle$. For Not-gate, through blue trajectory the quantum state pass from desired state $|0\rangle$ at some time $t_{*}<t_{f}$ and for Hadamrd gate at some other time the quantum state through blue trajectory passes from a vicinity of the desired state $|+\rangle$. For simulations, QuTiP^18^ Python package is used.

For flux-qubit $\lambda =-\frac{\pi }{2}$ rad, $\omega _{c}=32680005991$ rad/sec(or $f_{c}=5.2$GHz) in general and for NOT-gate we have the magnetic flux parameters $\phi _{0}=0.5422$ , $\phi _{dc}=-0.1355$ , and for Hadamard-gate we have current parameters as $\phi _{0}=0.3834$ , $\phi _{dc}=-0.1198$. Also we set the control final time equal to one nano-second. Figure 7 shows the result of simulations for this selection of initial and final states.

The results based on this qubit simulations for NOT and Hadamard gates are similar to phase qubit and only the state rotate a bit faster. Similar to previous qubits, here we also must solve the obtimization problem to obtain the time of arriving to desired states for each gate. The controlled propagator to be used for optimization with similar stategy to above calculations will be obtained as:

15 $$\begin{aligned} {U_{c_{pre}}=\cos (\frac{1}{\hbar }E_{L}\phi _{zpf}\phi _{e}t)I+i \mathrm {sin}(\frac{1}{\hbar }E_{L}\phi _{zpf}\phi _{e}t)\sigma _{x} } \end{aligned}$$

## Decoherence effect

**Figure 8:**
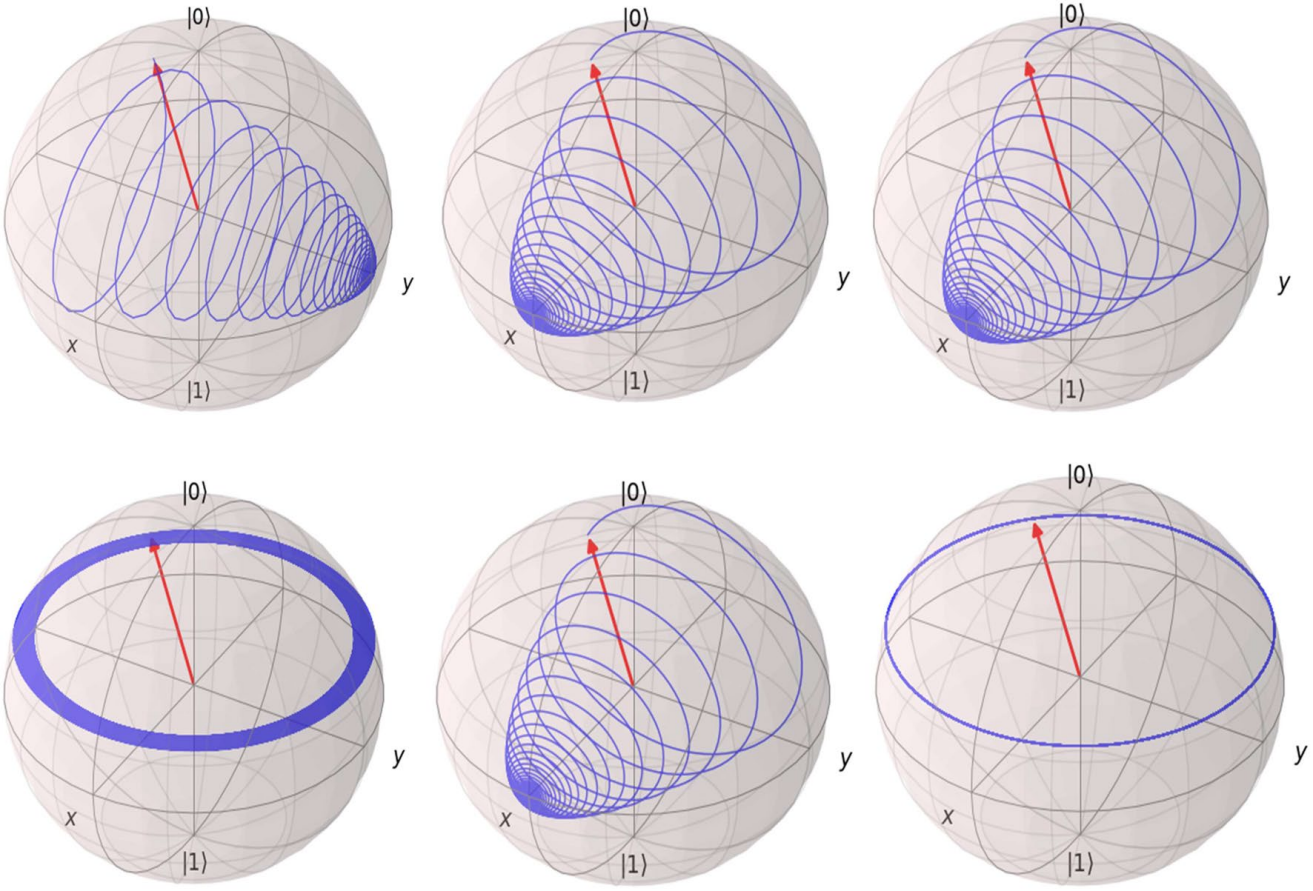
Decohrerence effect on evolution of fundamental superconductor qubits:charge qubit precise Hamiltonian (top-left), charge qubit approximate Hamiltonian (botton-left), phase qubit precise Hamiltonian (top-middle), phase qubit approximate Hamiltonian (botton-middle), flux qubit precise Hamiltonian (top-right) and flux qubit approximate Hamiltonian (top-right). Simulation of charge and flux qubits are carried for ten-thousand points and the phase qubits needs to be simulated with around ten-million point to show soft plots. Simulations are for one nanosecond. For simulations, QuTiP^18^ Python package is used.

The decoherence effect on quantum system can be described by Bloch-Redfield equation for desnity matrix as^28,29^. The general derivation of Bloch-Redfield master equation can be seen in^30^. The Bloch-Redfield master equation then is written as:

16 $$\begin{aligned} { \frac{d}{dt}\rho _{S}(t) = -i\omega \rho _{S}(t) + \sum _{E} {\mathcal {R}}_{SE} \rho _{E}(t)} \end{aligned}$$

in which ${\mathcal {R}}_{SE}$ is Bloch-Redfield tensor as a function of the noise-power spectrum $S(\omega )$ (i.e. quantum system-environment (SE)) and quantum system operator *A* through which the environment interacts with the quantum system. $\delta$ is a constant term. In this study, we consider the ohmic spectrum as: $S(\omega =0)=\gamma ,S(\omega >0)=\frac{\gamma \omega }{4\pi }$ that $\gamma$ is a dimensionless constant. We set $\gamma =0.5$ and $\delta =0.4\pi$, $A=\sigma _{z}$. The effect of decoherence on evolution of those three types of qubits with ohmic spectrum is depicted in Fig. 8. As we found, the decoherence effect on phase qubit has the same result both for precise and approximate Hamiltonian. About charge and flux qubits, the decoherence effect on precise system is completely different with respect to approximate system that has more regular effect. However, the modern control methods can compensate the decoherence effect and lead to good performance of approximate or precise system but the design for each case can be different. This is the reason why many reserchers use the approximate system and guide the qubit toward desired quantum state but with more control effort and different designs. The controller design for compensation of decoherence is out of scope of this research.

## Lyapunov superconductor qubit control

Let us compute the master equation for a charge-phase-flux qubit (parallel L-JJ-C circuit) for analysis and control purpose, the general controlled Hamiltonian is:

17 $$\begin{aligned} H=E_{c}(\hat{{N}}-n_{g})^{2}-E_{J}\cos ({\hat{\phi }})+\frac{1}{2}E_{L} ({\hat{\phi }}-\phi _{e})^2-\frac{\hslash }{2e}I_{g}{\hat{\phi }} \end{aligned}$$

let in first formulation get all of control signals in this charge-phase-flux qubit, by using $n_{g}=\frac{1}{2}C_{g}/eV(t), \phi _{e}=\phi (t),I_{g}=I(t)$, and with ${\hat{\phi }}=\phi _{zpf}\sigma _{x},\hat{{N}}=n_{zpf}\sigma _{y}$ , after computing the master equation, the following bi-linear differential equations arise:

18 $$\begin{aligned} {\dot{x}}(t)= & {} -\frac{n_{zpf}}{2e\hslash }E_{c}C_{g}V(t)z(t)\nonumber \\ {\dot{y}}(t)= & {} \phi _{zpf}(\frac{2}{\hslash }E_{L}\phi (t)+\frac{1}{e}I(t))z(t)\nonumber \\ {\dot{z}}(t)= & {} \frac{n_{zpf}}{2e\hslash }E_{c}C_{g}V(t)x(t) -\phi _{zpf}(\frac{2}{\hslash }E_{L}\phi (t)+\frac{1}{e}I(t))y(t) \end{aligned}$$

**Figure 9:**
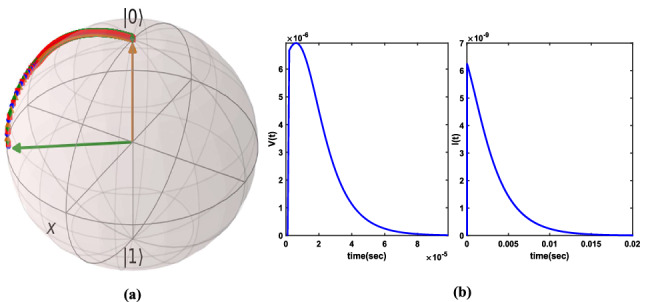
Trajectory of state control with Lyapunov control on Bloch sphere (**a**) and control signals (**b**). For simulations, QuTiP^18^ and Matplotlib^20^ Python packages are used.

It was found by this equation that in absence of external voltage, current or flux, the system always remains at initial state. Also, it is clear that for full control of states, it only needs control voltage *V*(*t*) and one of the flux or current signal. Let us set the external control flux signal be zero and controlling the system based on *V*(*t*), *I*(*t*). Equilibrium state is a state at which without control signals, system relaxes. For this system, all points are an equilibrium state. A control method for general quantum nonlinear systems is the Lyapunov function^4,31,32^. In Lyapunov method, we must find a positive definite scalar function and control signals must be selected such that the time derivative of Lyapunov function be negative definite, in this situation, it guarantes that the initial state stabilizes toward a desired state. Let $(x_{f},y_{f},z_{f})$ be the final state and $\vec {e}=(x_{t}-x_{f},y_{t}-y_{f},z_{t}-z_{f})$ be the error between state and final state, define the Lyapunov function as Euclidean norm of error $\gamma (\vec {e})=\frac{1}{2}|\vec {e}|^2$, hence: ${\dot{\gamma }}(\vec {e})=\dot{\vec {e}} \vec {e}^{T}={\dot{x}}_{t}(x_{t}-x_{f})+{\dot{y}}_{t}(y_{t}-y_{f})+{\dot{z}}_{t}(z_{t}-z_{f})$, then by this and Eq. (18) and setting $\phi _{t}=0$, it obtains: ${\dot{\gamma }}(\vec {e})=-\frac{1}{2e\hslash }n_{zpf}E_{c}C_{g}V(t)(x_{t}z_{f}-x_{f}z_{t})-\frac{\phi _{zpf}}{e}I(t)(y_{f}z_{t}-y_{t}z_{f})$, then by selecting the linear control voltage as $V(t)=\frac{2\alpha e\hslash }{E_{c}n_{zpf}C_{g}}(x_{t}z_{f}-x_{f}z_{t})$ and linear control current as $I(t)=\frac{\beta e}{\phi _{zpf}}(y_{f}z_{t}-y_{t}z_{f})$ for dimentionless ${\alpha _{1}} , {\alpha _{2}}>0$ then ${\dot{\gamma }}(\vec {e})=-{\alpha _{1}}(x_{t}z_{f}-x_{f}z_{t})^2-{\alpha _{2}}(y_{f}z_{t}-y_{t}z_{f})^2<0$ and the L-C-JJ qubit state will converge to final state and stabilizes. Simulation for initial state $\vec {r}_{0}=(4/9,-8/9,-1/9)$ and final desired state $\vec {r}_{f}=(0,0,1)$ is depicted in Fig. 9a. This simulation has 20000 samples and $dt=1\mu$sec, ${\alpha _{1}=\frac{1}{5}\alpha _{2}}=1\times 10^{10}$. As it is figured out from Fig. 9b, the voltage control is of order $\mu$v and the current control has order of nA. I mention that Lyapunov control guarantees to reach to desired state; however, it does not consider the control performance criteria. Hence for this aim the other control methods such as quantum optimal control or quantum reinforcement learning control can be developed.
